# An in vitro assessment of the physical properties of manually- mixed and encapsulated glass-ionomer cements

**DOI:** 10.1038/s41405-020-0040-x

**Published:** 2020-08-11

**Authors:** Lamis Al-Taee, Sanjukta Deb, Avijit Banerjee

**Affiliations:** 1 Department of Conservative and Aesthetic Dentistry, Baghdad College of Dentistry, University of Baghdad, Baghdad, Iraq; 2 Oral, Clinical & Translational Sciences, Faculty of Dentistry, Oral & Craniofacial Sciences, King’s College London, Guy’s Dental Hospital, London, UK; 3 Conservative & MI Dentistry, Faculty of Dentistry, Oral & Craniofacial Sciences, King’s College London, King’s College London, Guy’s Dental Hospital, London, UK

**Keywords:** Health care, Dentistry, Glass-ionomer cement

## Abstract

**Objectives:**

The last decade has seen a variety of modifications of glass-ionomer cements (GICs), such as inclusion of bioactive glass particles and dispensing systems. Hence, the aim was to systematically evaluate effect of mixing modes and presence of reactive glass additives on the physical properties of several GICs.

**Materials and methods:**

The physical properties of eight commercial restorative GICs; Fuji IX GP Extra (C&H), Ketac^TM^ Fill Plus Applicap (C&H), Fuji II LC (C&H), Glass Carbomer Cement and Equia® Forte Fil, capsulated versus manually mixed were assessed. 256 cylindrical specimens were prepared for compressive strength and microhardness, whilst 128 disc-shaped specimens were prepared for biaxial flexural strength tests. Fluid uptake and fluoride release were assessed. Data were analysed using one-way ANOVA and Games-Howell post-hoc tests (alpha = 0.05).

**Results:**

Both encapsulated GIC/RMGICs exhibited significantly improved mechanical properties in comparison to manually mixed equivalents, which in turn showed higher fluid uptake and early fluoride release (*p* < 0.05). The glass carbomer cement exhibited improved mechanical properties post ageing and evidence of mineral deposits were apparent in the microstructure.

**Conclusions:**

The mixing mode and inclusion of reactive glass additives in cements had a statistically significant effect on physical properties of the selected GICs-RMGICs.

## Introduction

Glass-ionomer cements (GICs) possess unique properties making them clinically attractive restorative materials. Advantages include the chemical adhesion to tooth tissues, low coefficient of thermal expansion, good tissue biocompatibility and fluoride ion release that potentially reduces the incidence of caries associated with restorations and sealants (CARS—secondary caries).^1,2^ Since its introduction at the early 1970s,^3^ GICs have been supplied as separate powder/liquid formulations with the relative proportions being dispensed by the operator. The problems identified with the manual-mixing of GICs in clinical practice were mainly related to variation in the powder: liquid (P:L) ratio. The difference in the powder packing densities achieved on filling the scoop and the way the bottle is held to dispense the drop leads to variation in the P:L ratio. Encapsulation allows maintaining the powder/liquid ratio and mixing regime is standardised hence the properties of the resultant GIC cements are not influenced by operator-induced variability.^4–6^ Glass carbomer cements® (GCP Dental, Mijlweg, Netherlands) a variation of GIC contain nano-sized glass particles, hydroxyapatite (Hap) and fluorapatite (FAp) as fillers that are expected to transform into an apatite-like material over time.^7^ The fine glass particles are thought to aid its dissolution and ultimate conversion to FAp and HAp promoting the remineralisation of demineralised tooth tissues.^8^ The high viscosity GICs with ultrafine glass particles, like Equia Fil, encourage greater cross-linking, which is believed to enhance mechanical properties, wear resistance and solubility as compared to conventional GICs.^9^ It has been previously reported that modifications in both powder and/or liquid components of various commercial GICs lead to major changes in physical properties of the cements. However, it is not clear if different mixing regimes influence the long-term properties of the resulting cements and how properties of cements with reactive fillers are influenced on in vitro ageing. This study reports the influence of two mixing regimes of six commercially available GIC/RMGICs and two newer GICs containing ultrafine glass or apatite on their physico-mechanical properties, which were tested under identical conditions for effective comparison of their properties. The null hypotheses proposed was that mixing regimes (mechanical vs. manually-mixing), the inclusion of reactive glass additives in GICs’ composition, and short-term ageing do not affect their physical properties.

## Materials and methods

The cements used in this study are listed in Table 1. The components of each material were mixed under controlled room temperature (23 ± 2 °C) and humidity (35 ± 5%), according to the manufacturers’ instructions.

**Table 1:** Capsulated (C) and manually -mixed (H) glass-ionomer cements (GICs) tested including the manufacturers’ details, composition and powder/liquid ratios.

Materials	Manufacturers	Code	Composition	P/L ratio
Fuji IX GP Extra	GC Corp., Japan	F9E (C),	CAFS-glass, PAA	0.4/0.12
Fuji IX GP Extra	GC Corp., Japan	F9E (H)	CAFS-glass, PAA	0.34/0.1
Ketac^TM^ Fill Plus Aplicap^TM^	3M Germany	KFPA (C)	CAFS-glass, PAMA 35–55%, TA 5–10%	0.36/0.1
Ketac^TM^ Fill Plus	3M, Germany	KFP (H)	CAFS-glass, PAMA 35–55%, TA 5–10%	0.32/0.1
Fuji II LC	GC Corp., Japan	F2LC (C)	CAFS-glass, PCA 5–10%, HEMA 25–50%, UDMA 1–5%, initiators, pigments	0.33/0.1
Fuji II LC	GC Corp., Japan	F2LC (H)	CAFS-glass, PCA 5–10%, HEMA 25–50%, UDMA 1–5%, initiators, pigments	0.32/0.1
Glass Carbomer Cement	GCP, The Netherlands	GC (C)	CAFS- glass 90% Apatite < 6%, Polyacids < 4%	–
Equia® Forte Fil	GC Corp., Japan	EF (C)	CAFS-glass, ultra-fine reactive glass, PAA	0.4/0.13

*CAFS-glass* calcium aluminofluorosilicate glass, *PAA* poly acrylic acid, *PAMA* copolymer of acrylic and maleic acid, *TA* tartaric acid, *PCA* polybasic carboxylic acid, *HEMA* 2- hydroxyethyl methacrylate, *UDMA* urethane dimethacrylate.

### Specimens preparation

Manually- mixed GICs (H) (F9E, KFP, and F2LC) were mixed according to the manufacturers’ recommended P/L mixing ratio, (Table 1). The bottle was tapped and shaken to unsettle the powder then dispensed on a glass slab and separated into two equal parts. The bottle containing the liquid was tipped onto its side, inverted and squeezed gently allowing the dispensing of a clear drop without air bubbles. Half of the powder was mixed with the liquid for 10 s, while the remaining powder was mixed for 25 s in accordance with manufacturers’ instructions. The encapsulated equivalents (C) of F9E, KFPA, and F2LC, and EF, and GC were dispensed in accordance with the manufacturers’ instructions. Cylindrical specimens (6.0 ± 0.1 mm height and 4.0 ± 0.1 mm diameter) were prepared for the compressive strength (CS and CM), and microhardness (MH) tests, while a stainless-steel mould with dimensions of 8.3 ± 0.1 mm diameter and 1.3 ± 0.1 mm thickness was used to prepare disc specimens for biaxial flexure strength determination (BFS). The mould was slightly over-filled with each material and sandwiched between two glass plates under constant pressure with standard load 500 mg over the mould to extrude any excess and provide parallel flat specimen ends.

F2LC (mechanical and manually mixed) specimens were photo-polymerised following the manufactures’ recommendations using a light-curing device (Model 503, Dentsply, Germany, light intensity 450 mW/cm^2^) for 30 s at each end of the cylindrical mould. A light intensity of 1400 mW/cm^2^ (CarboLED CL-01, GCP Dental, Vianen, The Netherlands) was used for the Glass Carbomer specimens. The specimens were removed from the moulds and stored in artificial saliva^10^ at 37 °C until testing at 1 and 30 days. The artificial saliva was refreshed once a week.

### Mechanical properties

#### Compressive strength

Sixteen cylindrical specimens of each commercial material were prepared for the compressive testing after 1 and 30 days. A universal testing machine (Instron model 5569, USA) with a 500 N load cell was used for testing the CS and modulus at a crosshead speed of 0.5 mm/min. The compressive strength was calculated using Eq. (1) and compressive modulus was determined from the linear region the stress-strain curve.

1 $$P = \frac{{4F}}{{\pi D^2}}$$

Where *F* was the load at fracture (N) and *D* was the mean specimen diameter (mm).

#### Hardness

The surface hardness (*n* = 16) was determined after 1 and 30 days using Knoop hardness test (Duramin10, Struers, Japan) using 50 gf load force for 10 s. The Knoop Hardness Numbers (KHN) were recorded as an average of 6 readings at randomly selected areas which are at least 1 mm far away from the adjacent indentations or the margin of the specimens.

#### Biaxial flexural strength

**Fig. 1:**
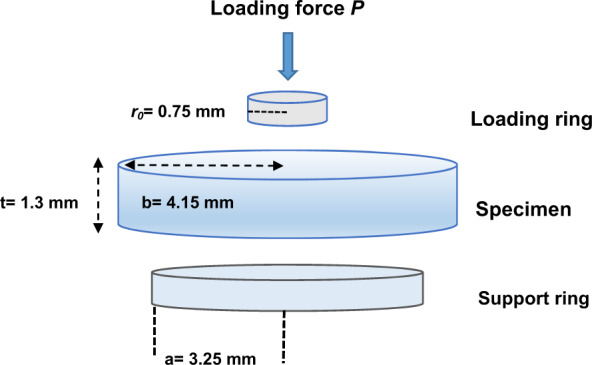
Biaxial flexural strength testing apparatus. (P) is the applied load at failure, (a) is the radius of support circle, (b) is the radius of disc specimen, (t) is the thickness of the disc specimen, and r_0_ is the radius of the ball used on the loading surface.

Sixteen disc-shaped specimens from each group were prepared and tested for BFS after 1 and 30 days. The specimen was placed centrally on a 6.5 mm diameter circular support in such a manner that the edge extended beyond the support by the same amount around the whole specimens. Then, this specimen was centrally loaded with a 1.5 mm diameter round ended indenter in a way that the area of maximum tensile stress was located at the centre of the lower face of the disc, (Fig. 1). The load was applied using a universal testing machine (Instron Model 5569, USA) at a crosshead speed of 0.5 mm/min until the specimens yielded or fractured.

The load at failure was obtained directly from the loading curves. The BFS value was calculated using the following equations, Eqs. (2)–(4):^11^

2 $$\sigma = \frac{{AP}}{{t^2}}$$

3 $$A = 3/\left( {4\pi } \right) \left[ {2\left( {1 + v} \right)\ln \left( {a/r_0^ \ast } \right) + \left( {1 - v} \right)\left\{ {\frac{{2a^2 - r_0^{ \ast 2}}}{{2b^2}}} \right\} + 1 + v} \right]$$

Where *P* is the applied load at failure, *v* is Poisson’s ratio (0.35 for GIC restoratives),^12^ *a* is the radius of support circle, *b* is the radius of disc specimen, *t* is the thickness of the disc specimen, and *r*_*0*_ is the radius of the ball used on the loading surface, as shown in Fig. 1:

4 $$r_0^ \ast \sqrt {\left( {1.6r_0^2 + t^2} \right) - 0.675t}$$

Where *r*_0_ is an equivalent radius of contact between the loading ball and the disc specimen, where loading is considered uniform.

### Fluid uptake

Fluid uptake was measured as percentage hydration using ten discs (8.3 mm diameter, 1.3 mm thickness) of each group immersed in artificial saliva. After storage in an incubator at 37 ± 1 °C, the surfaces were gently dabbed on a filter paper and weighed daily until 30 days. The percentage fluid uptake was determined using the following equation, Eq. (5):^13^

5 $${\mathrm{\% }}\,weight\,change = \frac{{Wt - W0}}{{W0}} \times 100$$

*Wt* is the weight at time *t*, and *W*_0_ is the initial weight of the specimen.

### Fluoride ion release

Fluoride ion release measurements were recorded up to 30 days (*n* = 10 per group). Specimens were immersed individually in capped polystyrene tube containing 2 ml of artificial saliva (pH 7.0) and stored at 37 °C. To avoid fluoride saturation, the storage medium was refreshed every 48 h up to 4 weeks. An equal volume (2 ml) of total ionic strength adjustment buffer (TISAB I BDH Ltd., Poole, England) was added prior to fluoride ion measurements, which increases the ionic strength of the solution to a relatively high level and hence increases the accuracy of the reading. Fluoride concentrations were recorded in ppm using a selective fluoride electrode (Cole Parmer 27502) connected to an ion analyser (OAKTON 510 ion series, Singapore). The amount of fluoride eluted from the GICs were converted into milligrams of F^-^ released per unit surface of area (mg F/cm^2^).^14^

### Scanning electron microscopy (SEM) and energy dispersion X-ray spectrometry (EDX) analysis of glass carbomer cement (GC)

Representative surfaces from the GC cement specimens were dried, carbon-coated, and examined using a scanning electron microscope (SEM, FEI Co. Ltd., Cambridge, UK) with an accelerating voltage: 10 kV, working distance: 20 µm, 10 µm, and 2 µm, and magnification: x2500, x10000 and x25000, respectively, coupled to an energy dispersive X-ray spectroscope (EDX) (EDAX Inc., 91 McKee Drive, Mahwah, NJ 07430 USA). Elemental analysis of the GC cement at 24 h and 30 days were carried out.

### Statistical analysis

One-way analysis of variance (ANOVA) with Games-Howell post-hoc tests (alpha level = 0.05) were used to assess differences in the physical properties among groups. The mean values were further compared by using Games-Howell post-hoc tests for multiple comparisons (IBM®, SPSS® statistics20, Chicago). Independent *t-*tests (*p* < 0.05) were used to compare the effect of time (1 and 30 days) on the mechanical properties of each tested material.

## Results

### Mechanical properties: compressive strength, microhardness and biaxial flexure

Auto-mixed GICs/RMGIC (C) (F9E, KFPA and F2LC) exhibited statistically significantly (*p* < 0.05) higher CS values in comparison to their manually mixed equivalents (H) after 24 h and 30 days (Table 2). EF(C), F9E(C) and KFPA(C) showed the highest values after both time intervals. F2LC (C) showed comparable values to F9E(C) and EF(C) post-ageing (*p* = 1.000). In contrast, the manually- mixed F2LC recorded the lowest CS (*p* < 0.05) that was comparable to GC at the early term (*p* = 1.000), and both GC and F9E (H) post storage (*p* = 0.352, 0.863, respectively). Short-term ageing reduced the CS of F9E(C&H) and EF(C) but enhanced it in KFPA(C&H), RMGICs (C&H) and GC (*t*-test, *p* < 0.05).

**Table 2:** Compressive strength (CS) and compressive modulus (CM) for the GIC-RMGICs after 1 and 30 days, shown as mean (SD), *n* = 8.

Groups	CS [MPa] 1 day	CS[MPa] 30 days	CM [GPa] 1 day	CM[GPa] 30 days
F9E (C)	205.2 (14.6)^*a^	181.9 (13.3)^*^d^	8.6 (0.3)^*gi^	8.8 (0.9)^ln^
F9E (H)	153.8 (11.2)^b^	141.8 (7.4)^e^^	5.0 (0.9)^h^	7.9 (0.6)^^mn^
KFPA (C)	193.1 (10.8)^*a^	210.1 (14.9)^*^f^	9.7 (0.5)^*g^	10.1 (0.5)^l^
KFP (H)	165.1 (13.7)^b^	171.1 (12.3)^d^	7.7 (0.7)^ij^	9.4 (0.5)^^ln^
F2LC (C)	169.8 (8.9)^*b^	181.9 (8.7)^*^d^	4.4 (0.2)^*hk^	5.8 (1.4)^^*o^
F2LC (H)	108.1 (12.6)^c^	125.8 (17.6)^^e^	2.4 (0.3)	2.7 (0.6)
GC	110.0 (6.4)^c^	134.6 (6.4)^^e^	3.6 (0.7)^k^	7.0 (0.6)^^mo^
EF	216.4 (18.1)^a^	186.6 (11.7)^^df^	7.1 (0.8)^j^	9.0 (0.7)^^l^

(*) significant difference between capsulated and hand-mixed GICs. (^) significant difference within each group after short-term ageing in artificial saliva (*t*-test, *p* < 0.05). Similar letters in columns indicate no significant differences among GICs (Games-Howell test post-hoc tests, an alpha level of 0.05).

The modulus values exhibited a similar trend. The encapsulated GICs (F9E, KFPA and F2LC) had higher modulus values however, over time, such differences were only significant in RMGICs (*p* < 0.001). Auto-mixed F9E and KFPA showed the highest initial compressive modulus among all groups. After ageing, these values are comparable to KFP (H) and EF (C) (*p* > 0.05). The modulus of all the cements appeared to increase post-ageing, however, it was only statistically significant in F9E (H), KFP (H), F2LC (C), GC and EF (*t*-test, *p* < 0.05).

The encapsulated GICs/RMGIC cements exhibited higher surface hardness than the corresponding hand-mixed cements at both time periods, Table 3. F9E (C) and EF (C) had the highest early KHN among all groups which was comparable to KFPA (C) post-ageing (*p* = 1.000, 0.923, respectively). RMGIC (C&H) showed lower hardness in comparison to the CGCs, however, the encapsulated group showed comparable early values to KFPA (C&H) (*p* = 0.783, 0.423, respectively), and F9E (H) and GC (*p* = 0.925, 0.085, respectively) post-ageing. All conventional GICs displayed an enhancement in microhardness after storage, however, it was only significant in F9E (H), KFPA (C&H) and GC. In contrast, RMGICs (C&H) exhibited a reduction in KHN over time, however, this decrease was not significant in the hand-mixed version (*p* = 0.064).

**Table 3:** Microhardness (MH) and biaxial flexural strength (BFS) for the GIC-RMGICs after 1 and 30 days (mean and (SD), *n* = 8).

Groups	MH [KHN] 1 day	MH[KHN] 30 days	BFS [MPa] 1 day	BFS[MPa] 30 days
F9E (C)	62.3 (4.4)^*a^	63.9 (4.5)^*f^	48.1 (6.2)^hi^	44.4 (5.9)^*jk^
F9E (H)	35.3 (2.5)^b^	39.3 (4.8)^^g^	40.7 (4.2)^h^	34.3 (4.9)^^l^
KFPA (C)	52.1 (2.9)^*c^	63.3 (4.1)^*^f^	70.0 (4.5)^*^	61.9 (2.1)^^m^
KFP (H)	44.4 (4.9)^d^	54.9 (2.9)^^^	42.9 (6.4)^hi^	60.7 (4.0)^^m^
F2LC (C)	49.4 (4.2)^*cd^	37.0 (2.6)^*^g^	135.8 (8.2)^*^	174.4 (7.0)^*^^
F2LC (H)	32.5 (2.9)^be^	28.0 (4.2)	122.8 (7.8)	91.5 (9.5)^^^
GC (C)	28.0 (2.6)^e^	40.6 (1.7^)^g^	27.2 (3.5)	38.0 (3.9)^^jl^
EF (C)	60.0 (3.0)^a^	61.1 (3.0)^f^	50.9 (3.7)^i^	50.7 (5.4)^k^

(*) significant difference between capsulated and hand-mixed GICs. (^) significant difference within each group after short-term ageing in artificial saliva (*t*-test, *p* < 0.05). Similar letters in columns indicate no significant differences among GICs (Games-Howell test post-hoc tests, an alpha level of 0.05).

The BFS values of the mechanically-mixed GICs/RMGIC were higher than the manually mixed version. However, the differences are not statistically significant in F9E after 24 h (*p* = 0.090) and KFP after 30 days (*p* = 0.919).

The BFS of the RMGIC (C&H) were significantly higher than the conventional GICs at both time intervals (*p* < 0.001). The encapsulated KFPA had the highest early BFS value among CGICs (*p* < 0.001), but after storage, both versions (C&H) of KFPA showed this trend. Short-term ageing showed a variable effect on the BFS of the tested GICs. Short-term ageing showed a variable effect on the BFS of the tested GICs with KFPA (H), F2LC (C) and GC showing a significant enhancement, whilst F9E (C), and EF remained unchanged, F9E (H), KFPA (C), F2LC (H) (*p* < 0.05) compromised post-ageing (Table 3).

### Fluid uptake

**Fig. 2:**
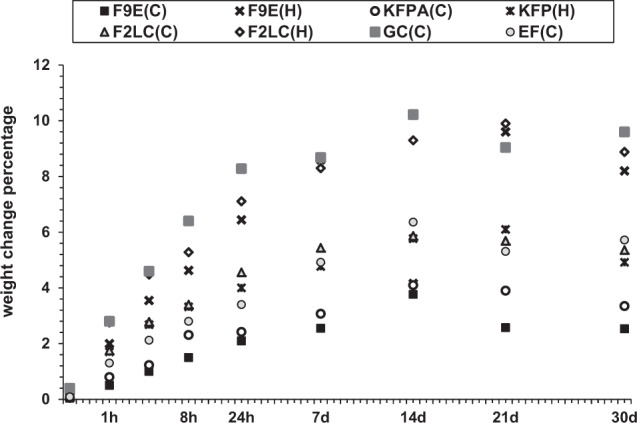
Fluid uptake of the GIC-RMGICs over 30 days. The manually-mixed GICs-RMGIC and GC showed higher hydration percentages than the encapsulated equivalents.

The manually- mixed GICs-RMGIC displayed higher fluid uptake than the encapsulated equivalent measured over 30 days (*p* < 0.05). F9E(C) exhibited the lowest artificial saliva uptake whilst the glass carbomer (GC) exhibited a higher rate and water content, whilst encapsulated RMGIC’s showed higher water uptake than their conventional GIC equivalents, as shown in Fig. 2.

### Fluoride release

**Fig. 3:**
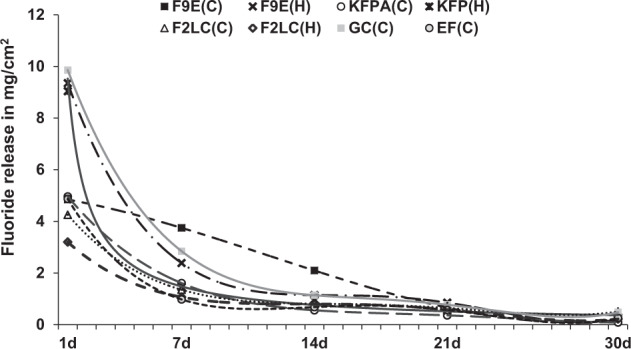
Fluoride release profile in mg/cm^2^ from the tested GIC-RMGICs up to 30 days. The early fluoride ion release was higher in the manually-mixed version of F9E and KFP, GC and the automixed of F2LC, however, the prolonged ion release was comparable in all investigated materials (*p* > 0.05).

The cements exhibited a similar pattern of fluoride release (in Fig. 3), that showed initial burst release in the first 48 h, followed by a decrease until reaching an asymptotic curve to equilibrium. In CGIC, the early fluoride release was higher in the manual mixed version (F9E and KFP) than the encapsulated equivalents, whilst a contrary trend was observed for F2LC. The early fluoride release was lower in RMGICs (C&H) in comparison to the conventional GICs. However, after 30 days, the amount of fluoride release was comparable in all investigated materials (*p* > 0.05).

### SEM-EDX analysis of glass carbomer cement (GC)

**Fig. 4:**
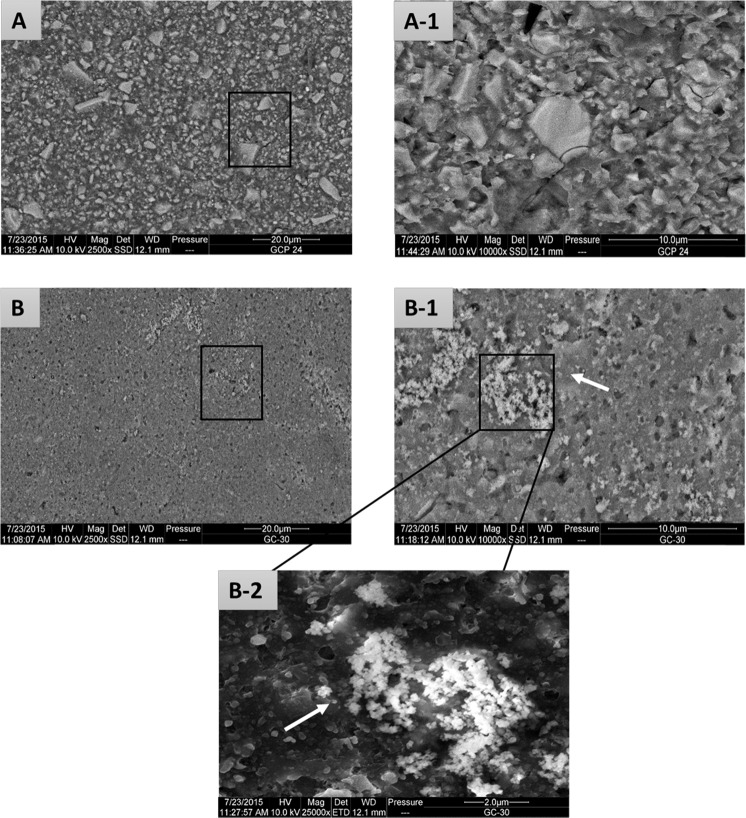
SEM micrographs of GC at different magnifications (x2500, and x10000). **a** GC-24 and **b** GC-30. White arrow in (B-1) showed the presence of mineral deposition on the surface of GC-30 which is more clearly observed at x25000 (B-2).

**Fig. 5:**
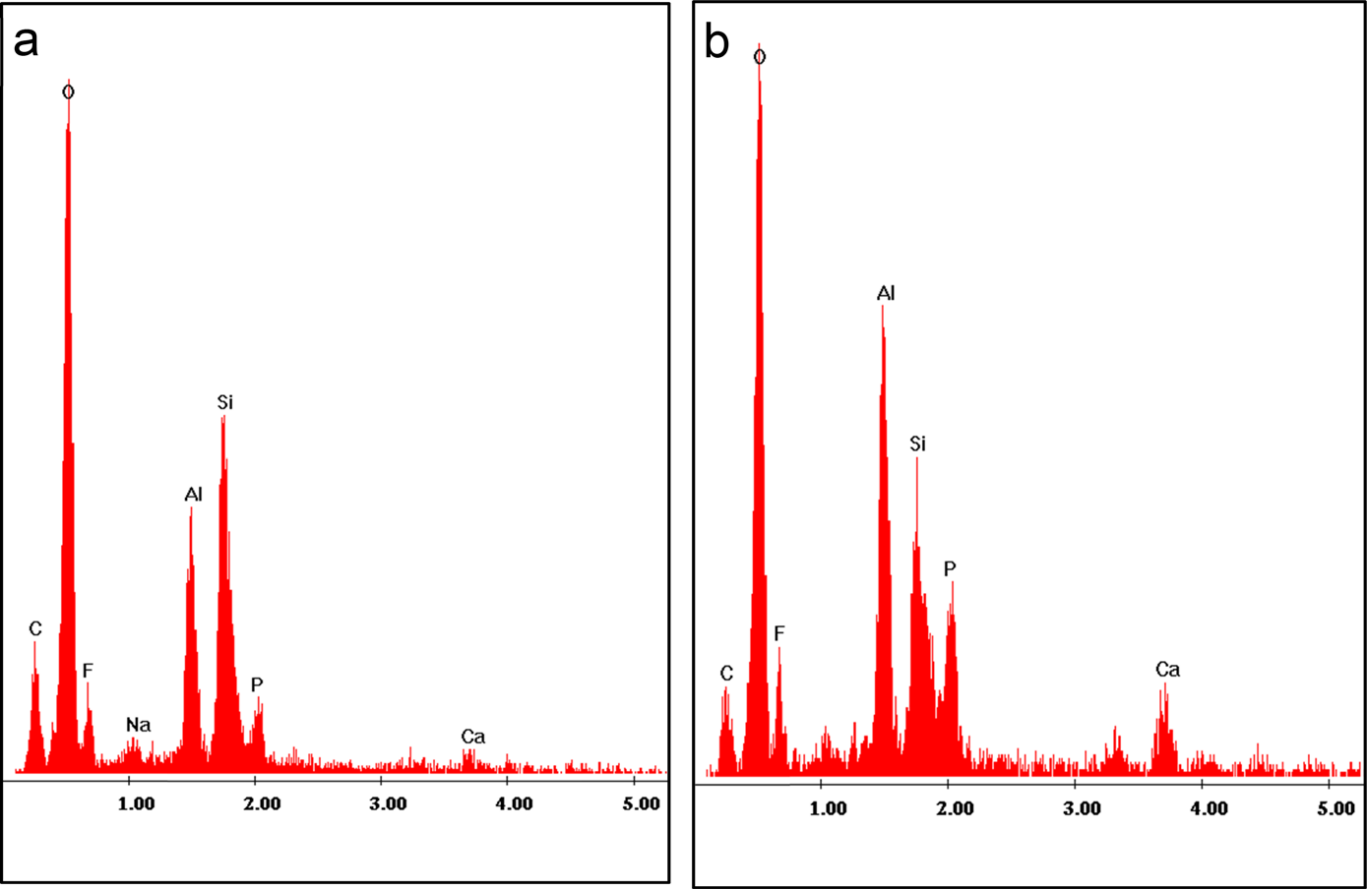
EDX analysis of glass carbomer cement GC after 24 h (**a**) and 30 days (**b**). There is a distribution of F, Si, Al, in addition to P and Ca, within their matrices. Blue arrows at (**b**) showed an increase in P and Ca ions peak in the cement post-ageing.

The glass carbomer cement showed dispersion of particles with varying size and shape whilst the specimens aged in artificial saliva for 30 days showed evidence of mineral deposits on the surface that were distinctly different from the particles with the cement. Mineral depositions were observed clearly on the surface of the GC-30 samples as shown in Fig. 4 (B-1 and 2). EDX analysis of GC-24 and GC-30 samples provided the distribution of F, Si, Al, in addition to P and Ca, within their matrices. Higher magnification SEM and EDX analysis of the mineral deposits show the presence of high amounts Ca and P within GC matrix post-ageing, Fig. 5b.

## Discussion

### Mechanical properties

#### Effect of mixing (mechanical vs. hand-mixing)

In line with previous findings,^5,15^ encapsulated GICs/RMGIC (C) exhibited higher compressive strength and modulus, microhardness and biaxial flexural strength than the hand-mixed equivalents (H) immediately and after 30 days, Tables 2, 3. The encapsulated versions eliminated the inaccurate dispensation prior to mixing and the mixing regime was standardised by mechanical mixing in accordance with the manufacturers’ instructions. Such mixing reduces porosity with more thorough wetting of the powder particles which enhance the setting reaction and thus the mechanical strength of the cement.^16^ Furthermore, the selected encapsulated materials have higher P/L ratios compared to the corresponding hand-mixed forms. This increases the initial viscosity and the homogeneity of the mix, thus improving the mechanical properties.^17^

The compressive strength values varied among groups as they are sensitive to variations in cements’ structure, the concentration of the reinforcing glass particles, and the presence of voids via air inclusion or inadequate wetting of the powder particles, which is associated with the mixing mode.^17,18^ Previous studies^19,20^ reported higher CS of the hand-mixed GIC’s, however, they utilised encapsulated GIC with lower powder content for a constant volume of liquid compared with the hand-mixed equivalent. In contrast, others^17,18,21^ reported higher compressive strength for the encapsulated GICs (190–210 MPa) than the manually mixed counterparts (130–160 MPa), in which the greater concentration of the glass filler combined with the reduction in air inclusion during mixing produce higher compressive strength.

Initially, the compressive moduli of all encapsulated GIC/RMGIC were significantly higher than the hand-mixed versions however, after cement maturation, the differences were not significant in CGICs. The conventional GICs showed higher compressive modulus values (5–9 GPa) than the RMGICs F2LC (C&H) (2.4–5.8 GPa), indicating that these materials would be more prone to brittle fracture with greater resistance to deformation and chipping than their methacrylate-modified counterparts.^21^

There is an agreement in the literature^22–24^ that the variations in microhardness of GIC’s are dependent on the maturation stage, setting reaction and the interactions with the storage medium. The early resistance is greatly influenced by the chemical composition, glass structure, the concentration and molecular weight of the polycarboxylic acid^22,23^ and the powder/liquid ratio.

Although, the beneficial effect of encapsulation of the GIC is not directly related to surface hardness, the mixing efficiency is expected to enhance the rate of setting reaction and hence result in faster increase of surface hardness with time^25^ and may even be responsible for higher KHN for the encapsulated systems. After 30 days storage in artificial saliva, the surface hardness was enhanced in all cGICs due to the ionic cross-linking and the formation of insoluble polysalt matrix over time.^19^ However, the completion of setting may not be a key factor for the property changes in RMGICs that exhibited lower hardness values after ageing. This is attributed to the lower amount of carboxylic acids as the conventional counterparts.^26^ Even though, the enhancement in the hardness of RMGICs on prolonged ageing is well supported in previous studies.^23,24^

The biaxial flexural strength test is recommended for brittle materials when subjected to multi-axial loads, since the maximum tensile stress occur within the central loading area. The RMGICs showed significantly higher BFS values after both time intervals in comparison to the conventional GIC, which is in agreement with literature findings.^21,23,27^ The resin components produce a homogeneous matrix of cross-linked poly-HEMA and polyacrylate salts which increases the resiliency and enable RMGIC to undergo flexure without fracturing while increasing the overall strength of the matrix.

#### Effect of composition of the GICs

The differences in the composition and P/L ratios of GICs have a direct influence on their physical properties. The higher powder/liquid ratio in EF, F9E (C) and KFPA (C) led to improved mechanical strength immediately and after storage. This fact is well supported by previous studies^22,28,29^ since the unreacted powder particles may act as reinforcing fillers within the matrix which impede crack propagation within the cement.

The inclusion of fine smaller-sized reactive glass particles coupled with higher P/L ratio in EF encouraged higher cross-linking with a possibility to act as strengthening fillers that increase the resistance of the cement to compressive loading.^9,29^ This leads to improved mechanical properties including compressive strength and modulus, biaxial flexural strength, and microhardness as compared to the other tested GICs. However, the inclusion of fine small-sized reactive hydroxyapatite and fluoroapatite particles (<6%) within the glass powder in GC did not show a beneficial effect in term of mechanical strength, which may be attributed to disruption of the cement forming process producing a cement with inferior mechanical properties.^30,31^

The presence of resin in GICs produces integrated network composite analogue composed of unreacted glass particles surrounded by a silica hydrogel, which are embedded in a cross-linked poly (alkenoic acid) -ion- resin copolymer. In accordance with previous findings,^6,32^ the elasticity of resin enables the RMGICs to undergo greater flexure without fracturing, hence increasing the BFS of the F2LC (C&H) and accounts for the lower modulus and microhardness. On the other hand, the rapidly formed polymer network between 2-HEMA and the pendant methacrylate groups might reduce the rate of the acid-base reaction and hinder the complete formation of poly-salt bridges,^6,32,33^ which might compromise the compressive strength of RMGICs as compared to their conventional counterparts. This trend is observed in the manually mixed version of F2LC which exhibited inferior compressive strength, modulus and microhardness than the conventional GICs except GC, however, the mechanical mixing enhanced the properties of F2LC that showed comparable values to many CGICs.

#### Effect of ageing

The setting reaction of GICs involve the reaction of the Ca^2+^ and Al^3+^ ions released from the aluminofluorosilicate glass with the water-soluble polymeric acid.^34^ During cement maturation the Al^3+^ ions that initially exist in four-coordination progressed to six-coordination state which enhances the mechanical properties to an extent. The evolution of strength of the GICs with time show distinct patterns of change since strengthening is attributed to the additional crosslinking and build-up of a silica gel phase,^20^ whereas weakening may result from the erosion and plasticising effect of water.^32^ The most noticeable enhancement in properties (CS, CM, MH and BFS) post ageing was seen in GC group. This enhancement is thought to be partially due to cement maturation, as well as the formation of ‘apatite’ like deposits arising from the dissolution of HAp within the GC matrix, which participate in hardening the cement. SEM observation supported these findings, as it showed dispersion of mineral deposits on the surface of aged cement Fig. 4 (B-1, and 2) associated with the presence of abundant quantities of Ca and P observed by EDX within the cement matrix after ageing (Fig. 5b). The results are consistent with studies of Moshaverinia et al.^29^ and Zainuddin et al.^8^ that revealed a dramatic rise in the mechanical properties of the cement containing HAp and FAp post-ageing as it produces stable hard, brittle material with a highly cross-linked polyacid salt matrix.

### Fluid uptake

Mechanical-mixing reduces air spaces between adjacent particles which minimises the porosity and enhances wetting of powder particles and thus improves the bulk properties of the resultant cement which might influence fluid diffusion into the matrix. Accordingly, all encapsulated GICs presented less fluid uptake than their hand-mixed equivalents over time, Fig. 2. In contrast, air voids that are generated by hand-mixing can accelerate the water uptake and solubility of these cements leading to less than optimal performance.^35,36^

GIC and RMGICs absorb water that is necessary for the acid-base setting reaction and ionic crosslinking. Water usually diffuses through the bulk of the cements via micro-voids or binding to the resinous groups which contain hydrophilic moieties such as HEMA (2-hydroxyethyl methacrylate).^37^ This might explain the higher fluid uptake observed in RMGICs in comparison to the conventional counterparts.

### Fluoride release

There is a marked difference in the amount of fluoride released in literature findings,^38,39^ and this study provides data that can be effectively used to compare fluoride release from the different materials as all conditions for release studies have been identically maintained. The release profile was characterised by an initial short-term burst release, followed by a prolonged and slowly elution (Fig. 3), which is similar to previous findings.^38,39^ As expected a mechanical mix yields a tightly bonded polyalkenoate matrix resulting in slow diffusion of fluoride from the cement matrix as initial elution depends on the ability of F^-^ ions to diffuse through cement voids, cracks and microporosities. Accordingly, auto-mixed GICs (F9E and KFPA) exhibited lower fluoride in comparison to their hand-mixed equivalents during the first 48 h. The fluoride ions in F2LC might be firmly encapsulated by resin matrix which might be responsible for the lower amount of initial release as compared to the conventional GICs, however, other studies^40,41^ have suggested that poly-HEMA can absorb sufficient water to enable diffusion of the fluoride ions.

## Conclusions

Encapsulated GICs and RMGICs (C) exhibited superior physical properties compared to their manually mixed equivalents (H) after 1 and 30 days. Encapsulated RMGIC showed satisfactory mechanical properties in comparison to the conventional GICs, while the hand-mixed RMGIC showed inferior mechanical properties. The presence of hydroxyapatite/fluorapatite (HAp/FAp) nanoparticles in glass carbomer cement did not enhance the early mechanical strength in comparison to other commercial GICs. However, there was a dramatic rise in the compressive strength, modulus, microhardness and biaxial flexural strength values post maturation may be associated with the precipitation of HAp within the matrix.

## References

[1] Moshaverinia A, Roohpour N, Chee WW, Schricker SR (2011). A review of powder modifications in conventional glass-ionomer dental cements. J. Mater. Chem..

[2] Nicholson J (2016). Adhesion of glass-ionomer cements to teeth: a review. Int J. Adhes. Adhes..

[3] Wilson AD (1978). The chemistry of dental cements. Chem. Soc. Rev..

[4] Fleming GJ, Marquis PM, Shortall AC (1999). The influence of clinically induced variability on the distribution of compressive fracture strengths of a hand-mixed zinc phosphate dental cement. Dent. Mater..

[5] Dowling AH, Fleming GJ (2009). Are encapsulated anterior glass-ionomer restoratives better than their hand-mixed equivalents?. J. Dent..

[6] Pameijer CH, Garcia-Godoy F, Morrow BR, Jefferies SR (2015). Flexural strength and flexural fatigue properties of resin-modified glass ionomers. J. Clin. Dent..

[7] Van RD, Davidson CL, De AG, Feilzer AJ (2004). In situ transformation of glass-ionomer into an enamel-like material. Am. J. Dent..

[8] Zainuddin N, Karpukhina N, Law RV, Hill RG (2012). Characterisation of a remineralising Glass Carbomer® ionomer cement by MAS-NMR spectroscopy. Dent. Mater..

[9] Sidhu SK (2011). Glass‐ionomer cement restorative materials: a sticky subject?. Aust. Dent. J..

[10] Eisenburger M, Addy M, Hughes JA, Shellis RP (2001). Effect of time on the remineralisation of enamel by synthetic saliva after citric acid erosion. Caries Res.

[11] Shetty DK, Rosenfield AR, Duckworth WH, Held PR (1983). A biaxial‐flexure test for evaluating ceramic strengths. J. Am. Ceram. Soc..

[12] Akinmade AO, Nicholson JW (1995). Poisson’s ratio of glass-polyalkenoate (“glass-ionomer”) cements determined by an ultrasonic pulse method. J. Mater. Sci. Mater. Med.

[13] Rojo L, Vázquez B, San Román J, Deb S (2008). Eugenol functionalized poly (acrylic acid) derivatives in the formation of glass-ionomer cements. Dent. Mater..

[14] Fukazawa M, Matsuya S, Yamane M (1987). Mechanism for erosion of glass-ionomer cements in an acidic buffer solution. J. Dent. Res..

[15] Molina GF, Cabral RJ, Mazzola I, Lascano LB, Frencken JE (2013). Mechanical performance of encapsulated restorative glass-ionomer cements for use with Atraumatic Restorative Treatment (ART). J. Appl. Oral. Sci..

[16] Nomoto R, Komoriyama M, McCabe JF, Hirano S (2004). Effect of mixing method on the porosity of encapsulated glass ionomer cement. Dent. Mater..

[17] Nomoto R, McCabe JF (2001). Effect of mixing methods on the compressive strength of glass ionomer cements. J. Dent..

[18] Fleming GJ, Kenny SM, Barralet JE (2006). The optimisation of the initial viscosity of an encapsulated glass-ionomer restorative following different mechanical mixing regimes. J. Dent..

[19] Pearson GJ, Atkinson AS (1991). Long-term flexural strength, of glass ionomer cements. Biomaterials.

[20] Williams JA, Billington RW (1991). Changes in compressive strength of glass ionomer restorative materials with respect to time periods of 24 h to 4 months. J. Oral. Rehabil..

[21] Mitra SB, Kedrowski BL (1994). Long-term mechanical properties of glass ionomers. Dent. Mater..

[22] Yap AU, Mudambi S, Chew CL, Neo JC (2001). Mechanical properties of an improved visible light-cured resin-modified glass ionomer cement. Oper. Dent..

[23] Kanchanavasita W, Anstice HM, Pearson GJ (1998). Long-term surface micro-hardness of resin-modified glass ionomers. J. Dent..

[24] Ellakuria J (2003). Effect of one-year water storage on the surface microhardness of resin-modified versus conventional glass-ionomer cements. Dent. Mater..

[25] De Moor RJ, Verbeeck RM (1998). Effect of acetic acid on the fluoride release profiles of restorative glass ionomer cements. Dent. Mater..

[26] Wilson AD (1990). Resin-modified glass-ionomer cements. Int. J. Prosthodont..

[27] McKenzie MA, Linden RW, Nicholson JW (2003). The physical properties of conventional and resin-modified glass-ionomer dental cements stored in saliva, proprietary acidic beverages, saline and water. Biomaterials.

[28] Behr M, Rosentritt M, Loher H, Handel G (2006). Effect of variations from the recommended powder/liquid ratio on some properties of resin-modified cements. Acta. Odontol. Scand..

[29] Moshaverinia A (2008). Effects of incorporation of hydroxyapatite and fluoroapatite nanobioceramics into conventional glass ionomer cements (GIC). Acta. Biomater..

[30] Yamamoto Y (1984). Study on hydroxyapatite-polyacrylic acid composite cement (hydroxyapatite-glass ionomer cement). Dent. Mater. J..

[31] Nicholson JW, Hawkins SJ, Smith JE (1993). The incorporation of hydroxyapatite into glass-polyalkenoate (“glass-ionomer”) cements: a preliminary study. J. Mater. Sci. Mater. Med.

[32] Cefaly DF, Mello LL, Wang L, Lauris JR, D’Alpino PH (2009). Effect of light curing unit on resin-modified glass-ionomer cements: a microhardness assessment. J. Appl. Oral. Sci..

[33] Eliades G, Palaghias G (1993). In vitro characterization of visible light-cured glass ionomer liners. Dent. Mater..

[34] Cattani-Lorente MA, Godin C, Meyer JM (1994). Mechanical behavior of glass ionomer cements affected by long-term storage in water. Dent. Mater..

[35] Kanchanavasita W, Anstice HM, Pearson GJ (1997). Water sorption characteristics of resin-modified glass-ionomer cements. Biomaterials.

[36] Cefaly DF, Franco EB, Mondelli RF, Francisconi PA, Navarro MF (2003). Diametral tensile strength and water sorption of glass-ionomer cements used in Atraumatic Restorative Treatment. J. Appl. Oral. Sci..

[37] Yap AU (1996). Resin-modified glass ionomer cements: a comparison of water sorption characteristics. Biomaterials.

[38] Verbeeck RM, De Moor RJ, Van Even DF, Martens LC (1993). The short-term fluoride release of a hand-mixed vs. capsulated system of a restorative glass-ionomer cement. J. Dent. Res..

[39] Thanjal NK (2010). Kinetics of fluoride ion release from dental restorative glass ionomer cements: the influence of ultrasound, radiant heat and glass composition. J. Mater. Sci. Mater. Med..

[40] Mitra SB (1991). Adhesion to dentin and physical properties of a light-cured glass-ionomer liner/base. J. Dent. Res..

[41] Forsten L (1995). Resin-modified glass ionomer cements: fluoride release and uptake. Acta. Odontol. Scand..

